# Adaptation and validation of the Adult Dispositional Hope Scale in the Ecuadorian context

**DOI:** 10.1186/s41155-023-00246-2

**Published:** 2023-01-23

**Authors:** Andrea M. Vinueza-Solórzano, Ronald Enrique Campoverde, Cecilia Alexandra Portalanza-Chavarría, Clarissa P. P. de Freitas, Claudio Simon Hutz, Ana Claudia Souza Vazquez

**Affiliations:** 1 Department of Psychology, Universidade Federal de Ciências da Saúde de Porto Alegre, Porto Alegre, RS 90050-170 Brazil; 2 Facultad de Ciencias Sociales y Humanísticas, Escuela Superior Politécnica del Litoral (ESPOL), Guayaquil, 090902 Ecuador; 3 Research Center, Universidad Espíritu Santo, Samborondón, 0901952 Ecuador; 4 Department of Psychology, Pontifical Catholic University of Rio de Janeiro, Rio de Janeiro, RJ 22451-900 Brazil; 5 Department of Psychology, Universidade Federal do Rio Grande do Sul, Porto Alegre, RS 90035-002 Brazil

**Keywords:** Dispositional hope, Validity, Reliability, Positive Psychology, Work Engagement, Burnout

## Abstract

This study aimed to evidence the validity and reliability of the Ecuadorian version of the Adult Dispositional Hope Scale, one of the most studied concepts of positive psychology. The adaptation process included translation and semantic and idiomatic validation. For content validation, an expert review and focus group were conducted. The questionnaire was applied to 2423 workers in Ecuador with a mean age of 37 years (SD = 9.04), and 65.6% were women. A confirmatory factor analysis was conducted to assess the validity of the scale’s dimensionality. The reliability and convergent and discriminant validity were also evaluated. In order to investigate the best solution for an Ecuadorian version of the Adult Dispositional Hope Scale, four structural models were assessed. The unidimensional solution was the most adequate structure for the scale. The internal consistency of the scale was adequate. The Adult Dispositional Hope Scale (ADHS) was developed to assess this positive psychological state and has been the most used tool in many contexts. To our knowledge, this study is the first to adapt the Adult Dispositional Hope Scale into the Ecuadorian context and evaluate its validity. The findings support its reliability, factorial, and construct validity in the Ecuadorian context. Furthermore, the results show that dispositional hope acts as a protective factor, promoting work engagement and preventing burnout.

## Introduction

Hope theory is one of the most studied concepts of positive psychology and is an important part of the theoretical framework for conceptualizing successful goal achievement (Cheavens et al., 2019; Feldman et al., 2009; Merolla et al., 2021). Traditionally, hope has been characterized as an emotion or cognition based on various epistemological foundations, such as social constructionism, behavioral models, or goal-directed emotion theories that link wishes with optimistic expectancies. In a combination of these theories, Snyder et al. (2005) reaffirmed the proposition made by Staats (1989) that hope is an affective cognition that occurs through the interplay between expectations and the desires behind them. Since these early studies, evidence for the main factors of hope can be observed, as goals (motivation to pursue a valued outcome), temporarily (future perspectives), and concerning emotions and cognitive coping strategies (Cheavens et al., 2019; Merolla et al., 2021).

The heuristic and integrative model proposed by Snyder et al. (1991)—the so-called Snyder Hope Theory—is the most widely adopted. Since that was published, hope has been studied as a positive and dispositional psychological state manifested mainly through a reciprocal cognitive (pathways) and motivational (agency) process (Merolla et al., 2021; Pacico et al., 2018; Pacico et al., 2013; Sun et al., 2012). The Adult Dispositional Hope Scale (ADHS) was developed by Snyder et al. (1991) to assess this positive psychological state. It has been validated in countries such as Italy (Alfieri et al., 2021), Spain (Galiana et al., 2015), France (Gana et al., 2012), Japan (Kato & Snyder, 2005), Germany (Krafft et al., 2019), Portugal (Marques et al., 2014), Brazil (Pacico et al., 2013), China (Sun et al., 2012), and Paraguay (Vuyk & Codas, 2019). Redlich-Amirav et al. (2018) reported that the ADHS is the most widely used tool (46%), followed by the Herth Hope Index (HHI, 16%). The ADHS is a general assessment of hope, whereas the HHI represents a clinical approach but lacks evidence of its reliability (Nayeri et al., 2020).

## Dispositional hope and mental health

Nowadays, there is robust evidence about the central play of dispositional hope as a relevant factor for mental health prevention and protection for individuals and populations in different cultures. Scientific advances in this field have shown that hope and its positive expectancies are protective factors for mental health in life and work and better therapeutic prognosis (Gallagher et al., 2020; Griggs, 2017; Gungor, 2019). Previous studies have generally shown that individuals with high levels of hope have the positive mental energy and the confidence to choose alternative paths when faced with problems in achieving goals in life and at work (Alves et al., 2018; Hellman et al., 2013; Snyder, 2000). Hope is a significant predictor of perceived satisfaction with life and work engagement and has positive and flourishing effects (Demirli et al., 2015; Ouweneel et al., 2012). Hopeful individuals can evaluate stressful situations as challenging rather than threatening (Vela et al., 2016).

Clinical research has demonstrated that lower levels of hope are significantly associated with depression, anxiety, and burnout at work (Bailey & Snyder, 2007; Feldman & Snyder, 2005; Leite et al., 2019; Masjedi-Arani et al., 2020). Hope plays a critical role in adaptation and recovery processes for mental disorders, in rehabilitation, and in reducing destructive interpersonal conflict and promoting pro-relational communication (Espinoza et al., 2017; Schrank et al., 2012). It is vital in counseling therapy and clinical interventions (Espinoza et al., 2017; Merolla et al., 2021; Schrank et al., 2012). Supportive interventions based on hope can bring benefits in preventing and reducing levels of anxiety and depression, including in patients with chronic diseases such as leukemia or other cancers, HIV-positive individuals, or armed forces veterans with chronic combat-related posttraumatic stress disorder (Alves et al., 2018; Schiavon et al., 2017). Hope serves as a personal resource in the workplace, where an individual is constantly subjected to emotional, physical, and psychological demands. It has the potential to promote a future-oriented motivational state that can help those individuals plan ways to achieve their goals and develop practical actions to succeed (Bakker & Demerouti, 2017; Leiter, 2021; Vazquez et al., 2019), and experience higher levels of work engagement (Hofkamp et al., 2020; Sarfraz et al., 2019). Also, there is evidence that hope acts as a protective factor against burnout at work (Passmore et al., 2020; Yotsidi et al., 2018). Empirical evidence confirms that the dispositional hope measured by ADHS maintains convergent validity with other variables (self-esteem, self-efficacy, satisfaction with life, anguish, hopelessness, depression, and anxiety) (Hutz et al., 2014; Yıldırım & Arslan, 2020).

## ADHS in the Spanish language

One of the criticisms in the field of Positive Psychological Assessment Measures (PPAM) is the “inconsistent factorial structures, varying ranges of internal consistency, and significant differences in their predictive capacity between cultures.” In our research, we identified three ADHS adaptations to the Spanish language, claiming that their evidence had adequate psychometric properties for its use in local and Latin American populations (Galiana et al., 2015; Vuyk & Codas, 2019; Vela et al., 2017, 2020). Nevertheless, questions should be posted in this PPAM proposed by all of them, mainly because of their validation on a particular population of students, with small and not representative samples, and the difference of their results on the factorial structure of ADHS.

Furthermore, we contend that, based on the sample and findings of these studies, a Latino/a population living and studying in the USA cannot be deemed representative of the Latin American population, as claimed by Vela et al. (2017, 2020).

Cultural differences influence people’s perceptions, emotions, achievements, motivations, and psychological well-being; thus, the ADHS must be examined and verified in diverse countries to reduce imprecision or misinterpretation (Merz et al., 2011). In the Ecuadorian context, people are exposed to a variety of environmental stressors working circumstances, political instability, and general social characteristics, whose context can lead in different ways to a positive outcome as hope, or negative aspects that can lead to harm their health and well-being (Merz et al., 2011; Ravallion, 2014; Vinueza-Solórzano et al., 2021).

## Factorial structure of hope

According to Snyder et al. (1991), hope represents a positive motivational state that is made up of two different ways of thinking about goals: pathways and agency (Muyan-Yılık & Demir, 2020). Pathway thinking reflects the perception of personal skills to generate routes to desired goals. Furthermore, the authors highlight that the subjective experience of hope does not depend on concrete and actual routes but rather the perceived tracing of necessary effective routes. The agency is conceptualized as the perception of people’s ability to initiate and continue on the selected paths to achieve their goals (Sharpe et al., 2019). In the hope theory, the combination of these components makes it possible to achieve the desired goal; however, a lack of either of them significantly reduces the likelihood that an individual will achieve their objective (Feldman & Snyder, 2005). Notably, the simple existence of agency (motivation) or pathways or routes (planning) is not enough to manifest hope, so these two factors likely interact throughout the goal-searching process.

The ADHS’ factorial structure is not clearly defined as proposed initially (Snyder et al., 1991) since studies also evidenced unidimensionally (Brouwer et al., 2008; Pacico et al., 2013) a two first-order factor with a second-order model (Alfieri et al., 2021; Gana et al., 2012; Kato & Snyder, 2005; Marques et al., 2014) and a bifactorial model (Sun et al., 2012; Vuyk & Codas, 2019). Considering the lack of consensus on this topic, with contradictory results, our research aims to contribute with evidence in this field. Also, in an examination of the unidimensional model, Gomez et al. (2015) suggest that the sense of pathways and agency of the Snyder Hope Theory model is reciprocal, of joint operation, and that although the two dimensions are correlated, they are not synonymous. The authors analyzed the distinction between second-order and bifactorial models of the Dispositional Expectancy Scale. They demonstrate that the bifactorial model has an advantage over the second-order model in that it allows relationships between items, not only between factors, to be evaluated.

Among these studies, it is plausible that Galiana et al. (2015) evidence of a unidimensional model for the ADHS applied to a Spanish population, which showed good psychometric properties, will be confirmed in the Ecuadorian context. Although Vuyk and Codas (2019) argue for a bifactorial model with second-order factors, their data has more sample questions. On the other hand, the study of Vela et al. (2017, 2020) reduced the ADHS to a different scale with 5 items, which is not what we are studying. Hellman et al. (2013) concluded from a meta-analysis that when an eight-item response format was used for dispositional hope assessment, internal consistency scores were higher (*α* = .82) than with the original four-item format (*α* = .77). They also estimated acceptable average reliability through a test-retest process that suggests stability over time and in different situations.

There is a lack of studies on hope in Ecuador despite the international findings showing the importance of hope for mental health, including the data collected in Spanish-speaking populations. At the same time, a validity and reliability analysis process through the evaluation of ADHS’s psychometric properties must be improved regarding more representative adult samples and the factorial analyses. These findings would allow scientists and practitioners to better evaluate hope as a protective factor and its role in the practice.

We intend to contribute to the field of the PPAMs built on valid and reliable measures of the hope construct so that it can be used with confidence in a particular context and for cross-cultural comparisons. The main objective of this study was to investigate the psychometric properties of ADHS in Ecuador. The dimensionality of the Ecuadorian version of ADHS was evaluated through confirmatory factor analysis. Its reliability was measured through ordinal Cronbach’s alpha (*α*), omega (*ϖ*), composite reliability (CR), and average variance extracted (AVE) (Valentini & Damásio, 2016; Viladrich et al., 2017).

External validity was investigated for the relationships between hope and both work engagement and burnout, because evidence shows that hope may act as a factor that promotes well-being at work (Demirli et al., 2015; Passmore et al., 2020; Yotsidi et al., 2018; Querido & dos Anjos Dixe, 2016). In order to deepen the evidence of the validity of the scale, we investigated if the Ecuadorian version of ADHS would be invariant for gender.

## Methods

### Data collection

The survey was conducted online during September and October 2020. Participants answered the instruments through an online survey published on the researchers’ social networks through the QuestionPro tool. Participants needed to agree with the consent form to access the instruments. The consent form presented the importance, objectives, and voluntary and confidential nature of the present study.

### Participants

A convenience sampling method was used to apply the instrument on professionals in Guayaquil, Ecuador, with 2423 participants, with a mean age of 37 (SD = 9.04, the age range was from 20 to 73 years) and 65.6% (*n* = 1588) women. The inclusion criteria of the participants were being over 18 years old and being Ecuadorian. Only 3 participants showed missing data, which were not imputed.

### Measures and instruments

#### Dispositional hope

This study was carried out for the Ecuadorian setting on the Snyder et al. (1991) ADHS initially validated with a unidimensional structure model on a theoretical assumption basis for future studies’ prospects. The scale has a total of 12 items: four pathway items (items 1, 4, 6, and 8), four agency items (2, 9, 10, and 12), and four filler items (3, 5, 7, and 11). Agency items measure the level of motivation to obtain goals, and pathways evaluate the perceived ability to identify and develop routes to achieve a goal. Filler items are used to reduce participants’ response bias. Answers are given on a 5-point Likert-type scale, in which 1 corresponds to “totally false” and 5 indicates “totally true.” The internal consistency of the original instrument was adequate (Cronbach’s alpha, *α* = .71–.84).

#### Burnout

The Burnout Assessment Tool (BAT) was developed to report burnout as a general score and to assess each one of its core dimensions (exhaustion, mental distance, cognitive impairment, and emotional impairment) (Schaufeli, De Witte, & Desart, 2020). The BAT version with 23 items (BAT-23) assesses exhaustion with eight items (items 1 to 8), mental distance is evaluated by five items (items 9 to 13), cognitive impairment is evaluated by five items (items 14 to 18), and emotional impairment is evaluated by five items (items 19 to 23) (Schaufeli, Desart, & De Witte, 2020). Participants respond to items on a scale of 1 (“never”) to 5 (“always”). The BAT-23-Ecuadorian validation study showed the excellent value of internal consistency (Cronbach’s alpha, *α* = .94 (.93–.94) (Vinueza-Solórzano et al., 2021). The satisfactory goodness-of-fit indexes of BAT evidence the suitability of the BAT to assess burnout in the Ecuadorian population [CFI = .96, TLI = .96 and RMSEA = .076 (90% C.I., .074–.078)]. Furthermore, the results demonstrated that BAT was a reliable instrument to assess the general levels of burnout (*α* = .95, *ϖ* = .90, and CR = .98) and its dimensions exhaustion (*α* = .93, *ϖ* = .90, and CR = .93), mental distance (*α* = .85, *ϖ* = .76, and CR = .87), cognitive impairment (*α* = .95, *ϖ* = .91, and CR = .67), and emotional impairment (*α* = .93, *ϖ* = .88, and CR = .93).

#### Work engagement

Engagement at work was assessed with the short version of the Utrecht Work Engagement Scale, translated into Spanish, consisting of nine items (Schaufeli et al., 2006). The items were answered on a 7-point Likert scale, ranging from 1 (“never”) to 7 (“always”). The UWES goodness-of-fit indexes in the Ecuadorian population show that this instrument is suitable for evaluating engagement [CFI = .97, TLI = .96, RMSEA= .214 (90% C.I., .205–.221)]. Based on the values of the consistency indices, it is evidenced that the UWES can also be considered reliable to assess the levels of engagement in the Ecuadorian population (*α* = .95, *ϖ* = .93, CR = .96).

### Procedures

#### Translation and adaptation

The ADHS translation process was carried out following international methodological standards for adapting instruments to other cultures (AERA et al., 2014). The original ADHS was translated from English into Spanish by a certified translator with knowledge of English-speaking culture, thus favoring conceptual rather than literal translations. The translator was instructed on the Ecuadorian version. Content validity was assessed by five researchers to maintain the conceptual similarity of the items in the process and to maximize the equivalence between the original and the specialization in psychometrics or positive psychology. The researchers evaluated that the ADHS Ecuadorian version was adequate and did not suggest modification. The ADHS Ecuadorian version was reviewed in a focus group (*n* = 8) that examined idiomatic and semantic understanding of the items. Based on this feedback, no significant changes were made. Then, the instrument was translated back to English by a different English-speaking translator with knowledge of the construct. The comparison of the semantic and comprehension of the items’ meaning of the original ADHS and the Ecuadorian version by the authors of the present study evidence that the translated version was adequate.

#### Ethical statement

The Scientific Committee of Research and Publications from the University Espiritu Santo, Samborondón, Ecuador, approved the study of the project “Factores de Bienestar Laboral en Ecuador,” code no. 2021-ECON-002. The Institutional Review Board registration no. IRB00012254 was approved for use until February 28, 2022, for the University of Specialties Espiritu Santo.

All participants gave written informed consent by signing the online informed consent form, where they were informed that participation was anonymous and voluntary.

#### Data analysis

Four models were tested in this study: the unidimensional model (model 1), two first-order factors with second-order factor (model 2), the bifactor structure of two oblique S-factors and one G-factor (model 3), and two oblique first-order factors (model 4). These models (models 3 and 4) were tested to demonstrate the high correlation of the two dimensions (Agency and Pathways) that describe the phenomenon of dispositional hope and to evidence that the variance of the observed variables would constitute the latent variable comprehended as hope.

All confirmatory factor analyses (CFA) were performed using the weighted least squares mean and variance-adjusted (WLSMV) estimation method, as it is a sufficiently robust estimation method for ordinal data. The goodness-of-fit of the ADHS was assessed using the following fit indices: chi-square/degrees of freedom (*χ*^2^/df) ratio, the Comparative Fit Index (CFI), the Tucker-Lewis Index (TLI), and the root mean square error of approximation (RMSEA). According to the guidelines used (Brown, 2015), an acceptable fit is indicated if *χ*^2^/df is less than 3, the CFI and TLI values are more significant than.95, and the RMSEA values are less than .08, with a 90% confidence interval not greater than .10. Modification indices (MI > 50.00) were analyzed to identify the sources of specific problems in the four models evaluated (Brown, 2015). A chi-square difference (*χ*^2^) analysis was conducted to assess if the goodness-of-fit indices of models 1–4 were significantly different for the Ecuadorian version of the ADHS.

The invariance between male and female ADHS scores was assessed through multigroup CFA. To evaluate the measurement invariance, the configural, threshold, metric, scalar, and uniqueness invariances were compared based on a hierarchical method, in a way that the more restricted model was compared to the less restricted model. Based on values of CFI difference (ΔCFI) and RMSEA difference (ΔRMSEA) between the models, ADHS measurements are defined as invariant if ΔCFI is less than 0.01 (ΔCFI < 0.01) and ΔRMSEA is less than .015 (ΔRMSEA <0 .015) (Putnick & Bornstein, 2016).

The reliability of the scale was assessed using ordinal Cronbach’s alpha (*α*), omega (*ϖ*), composite reliability (CR), and average variance extracted (AVE) (Valentini & Damásio, 2016; Viladrich et al., 2017). For constructs with categorical indicators, the ordinal *α*, *ϖ*, CR, and AVE values were calculated from polychoric (polyserial) correlations and not from Pearson correlations (Valentini & Damásio, 2016). For the scale to be considered reliable, AVE values should be greater than .50 (Fornell & Larcker, 1981), and ordinal *α* should be greater than .70 (Viladrich et al., 2017).

Evidence based on the expected relationship with external variables was evaluated through convergent validity, using work engagement and burnout and its dimensions (exhaustion, mental distance, cognitive impairment, and emotional impairment). We expected that higher levels of hope would be positively associated with work engagement and negatively with burnout and its dimensions.

The discriminant validity of ADHS was evaluated by calculating if the AVE value of each construct (hope, work engagement, burnout, and its dimensions) was higher than the square correlations of the constructs with each other (Fornell & Larcker, 1981). All the analyses described in this study were performed in R Studio version 1.4.1717 (R Core Team, 2021), using the packages psych (Revelle, 2020) and lavaan (Rosseel, 2012) for structural equation modeling and MBESS (Kelley, 2020) to evaluate the reliability indices of the scale.

## Results

### Dimensionality

Descriptive statistics showed that participants answered the maximum range in all items. The distributional properties of most ADHS items presented satisfactory univariate normality (Table 1). Mardia’s multivariate skewness value was 32.7 (*p* < .001), and the kurtosis value was 177.6 (*p* < .001).

**Table 1:** ADHS items’ distributional properties

Item	M	SD	Min	P25	Mdn	P75	Max	sk	ku
1	4.4	.9	1	4	5	5	5	− 1.8	3.0
2	4.6	.9	1	4	5	5	5	− 2.5	6.3
4	4.5	.9	1	4	5	5	5	− 2.0	3.6
6	4.3	1.0	1	4	5	5	5	− 1.7	2.4
8	4.4	.8	1	4	5	5	5	− 1.6	2.6
9	4.5	.8	1	4	5	5	5	− 1.9	3.7
10	4.2	.9	1	4	4	5	5	− 1.3	1.9
12	4.5	.8	1	4	5	5	5	− 1.7	3.3

*M* mean, *SD* standard deviation, *Min* minimum, *P25* percentile 25, *Mdn* median, *P75* percentile 75, *Max* maximum, *sk* skewness, *ku* kurtosis

**Fig. 1:**
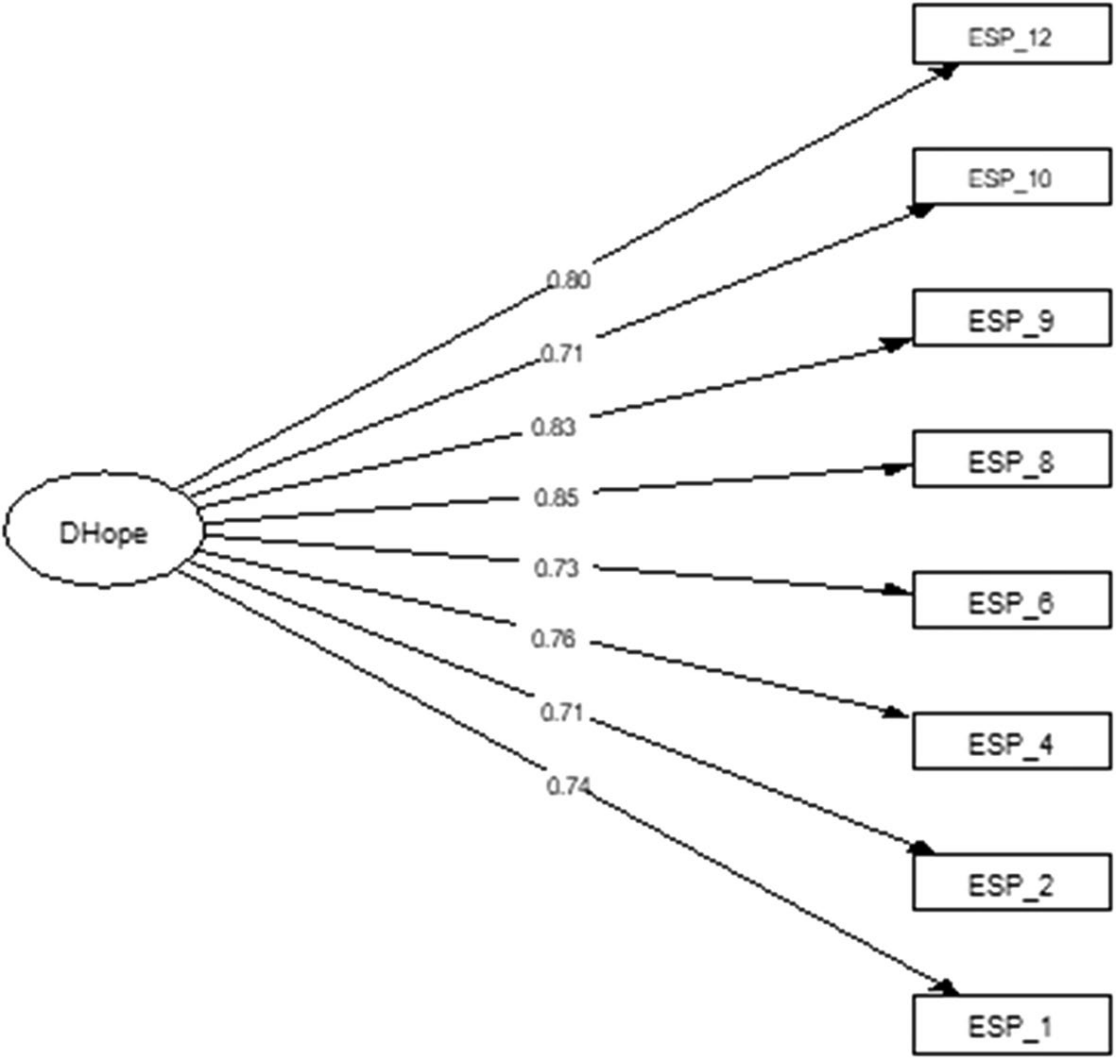
Unidimensional structural factor model for the Adult Dispositional Hope Scale

In order to investigate the dimensionality validity evidence, four CFA were performed. The unidimensional model (model 1) aimed to assess if the eight items would show high factorial loading on the general factor of hope. The unidimensional model (model 1) evaluated if a single factor could explain most of the variance of the observed variables (Brown, 2015; Dunn & McCray, 2020; Gustafsson & Åberg-Bengtsson, 2010). The results of the first CFA, which evaluated the unidimensional solution (model 1) for the ADHS, showed adequate goodness-of-fit indices, excluding the value of *X*^2^/df and RMSEA that was too high. Based on the high values of *X*^2^/df and RMSEA, modification indices were analyzed. Modification indices suggested the inclusion of error covariances for the following item pairs: 1-2 (MI = 831.88), 10-12 (MI = 469.12), and 10-12 (MI = 918.57) (Table 2). All items in this model showed factor loadings greater than .70 (Fig. 1).

**Table 2:** Confirmatory factor analysis of the hypothesized models

	df	X^2^	***P***	X^2^/df	CFI	TLI	RMSEA
**Model 1—**unidimensional solution	20	781.93	> .001	39.10	.945	.923	.125 [90%, .118–.133]
**Model 1**—unidimensional solution (E1–E2, E8–E9, and E10–E12)	17	251.12	> .001	14.77	.983	.971	.076 [90%, .068–.084]
**Model 2—t**wo first-order factors and second-order factor solution	18	269.10	> .001	14.95	.982	.972	.076 [90%, .068–.084]
**Model 3—b**ifactor solution	11	231.32	> .001	21.02	.984	.958	.092 [90%, .082–.102]
**Model 4—**two first-order oblique factors	19	760.41	> .001	40.02	.946	.921	.127 [90%, .119–.135]
**Model 4—t**wo first-order oblique factors (E1–E2, E8–E9, and E10–E12)	16	181.99	> .001	11.37	.988	.978	.066 [90%, .058–.075]

Model 2 assessed if ADHS would obtain two first-order factors with a second-order factor structure. The two first-order factors with a second-order model (model 2) assume that the two distinct factors are indicators of one general underlying factor (i.e., dispositional hope), which is thought to give rise to the correlation existing between the two first-order factors (agency and pathways). The first-order factors aimed to explain the variance of the observed variables, while the second-order factor seeks to explain the variance shared by the first-order factors (Brown, 2015; Dunn & McCray, 2020).

This second model showed excellent goodness-of-fit indices, disregarding the high value of the *X*^2^/df (Table 2). In order to avoid the inclusion of variance that cannot be explained by the theoretical propositions of the model, the parameters observed in the modification indices were not included in the model. The modification indices are parameters that contribute to the improvement of the fit of the model; however, they add a variance that cannot be explained by the relations proposed in the theoretically proposed model. Based on the model goodness-of-fit indices, it was understood that the inclusion of the parameters observed in the modification indices would not contribute to the explanation of the model.

**Fig. 2:**
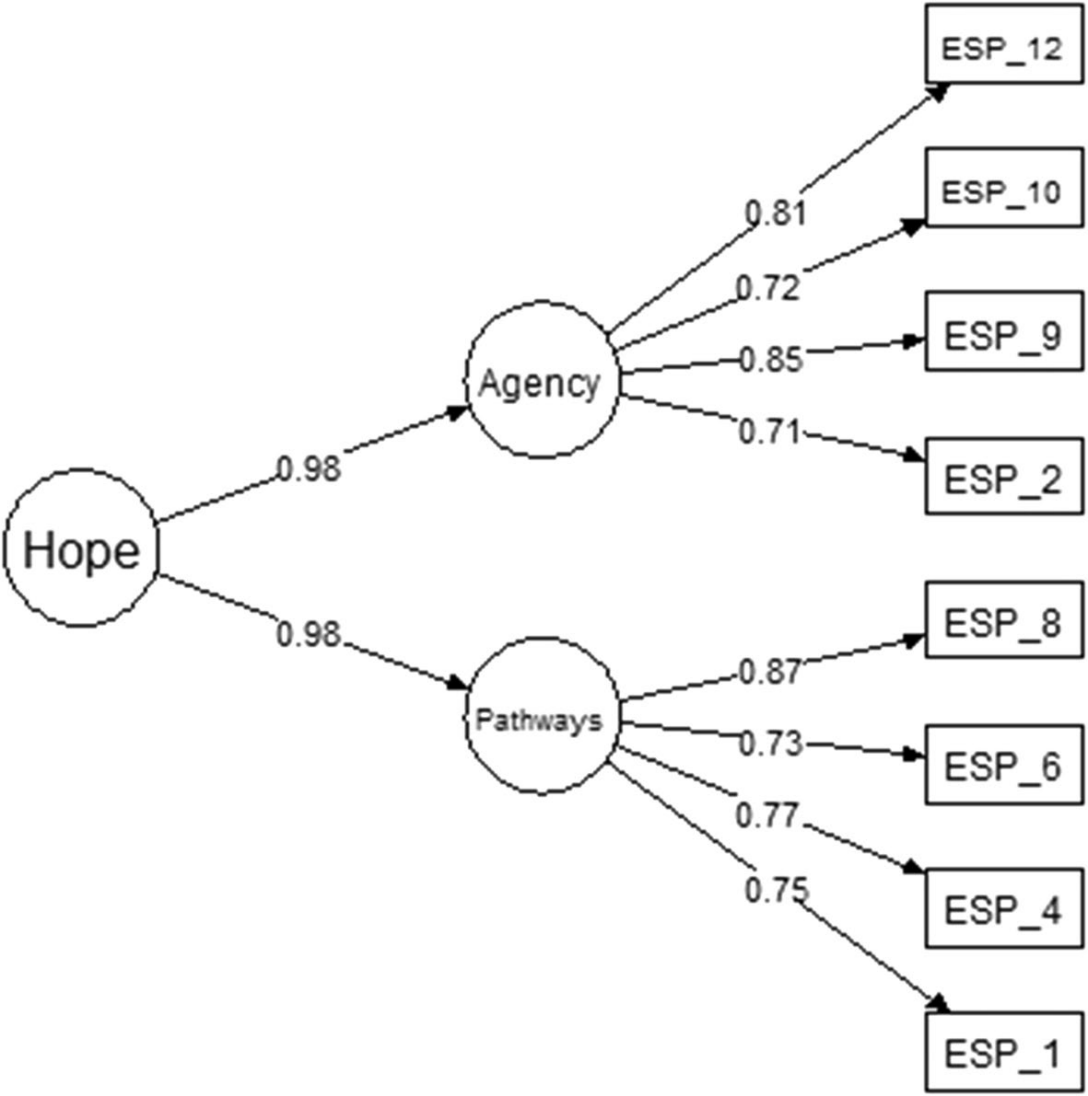
Two first-order factors and a second-order factor structural factor model for the ADHS

Despite the goodness-of-fit indices of model 2, this solution is questionable because it underestimates the direct effect of the second-order factor and the error variances (Kline, 2015). As shown in the path diagram of model 2 (Fig. 2), the two first-order factors were loaded on the second-order factor of general hope with a factorial loading greater than .95. This finding shows a lack of differentiation between the first-order and second-order factors, particularly between the dispositional hope and pathways dimensions. By analyzing the item loading in these two first-order factors, we determined factor loadings greater than .70. Based on that finding, we concluded that model 2 should be discarded.

Model 3 investigated a bifactorial model composed of two oblique S-factors and one G-factor. The bifactorial model proposes that the variance of observable variables can be explained simultaneously by a general factor (G-factor), which is composed of all items in the scale, and by at least two first-order orthogonal or oblique factors (*S*-factors), which of them are composed by the items of their respective dimensions. In a bifactor model, items load simultaneously on the G-factor and their respective S-factors (Dunn & McCray, 2020; Reise et al., 2018). In the present study, the G-factor was hope and the S-factor was agency and pathways. The bifactor model of two oblique S-factors and one G-factor (model 3) showed adequate goodness-of-fit indices, except for the value of *X*^2^/df that was higher than recommended (Table 2). As expected, pathways and agency were positively associated. In this model, the parameters observed in the modification indices were not included, because the addition of the unique variance of the modification indices would not improve the comprehension of the bifactor structure of the Ecuadorian version of ADHS.

**Fig. 3:**
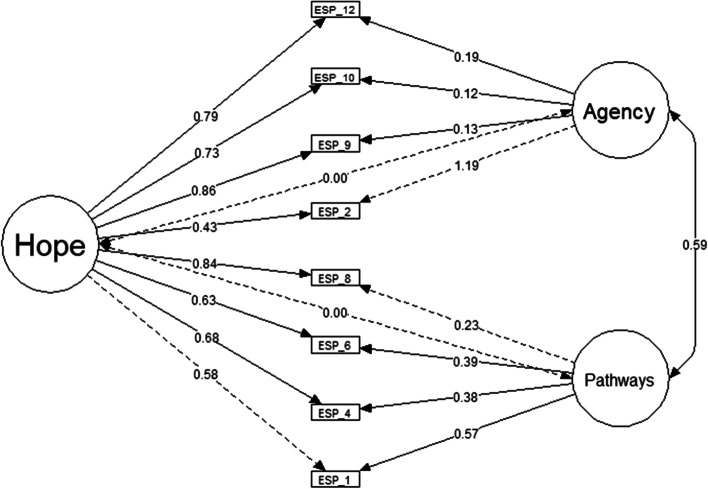
Bifactor structural factor model for the ADHS

Although model 4 presents adequate goodness-of-fit indices, the item factor loadings showed that the variance could be predominantly explained by the G-factor (hope) and less by the S-factors (agency and pathways; Fig. 3). Variance of items 8, 9, 10, and 12 were mainly explained by the G-factor that constitutes hope. Despite items 1, 4, and 6 being explained by the G-factor (hope) and the S-factor (pathways), the items showed a higher loading factor on the hope factor. Item 2 obtained a higher loading factor on the S-factor that constitutes pathways in comparison with the G-factor (hope). Even so, the loading factor of item 2 in pathways higher than 1.0 may be interpreted as a Heywood case, indicating the misfit of the bifactor model (Farooq, 2022). These findings evidence that the bifactor model in which a general hope factor explains part of the variance, with pathways and agency explaining the remaining additional variance, is inadequate for the Ecuadorian version of ADHS.

**Fig. 4:**
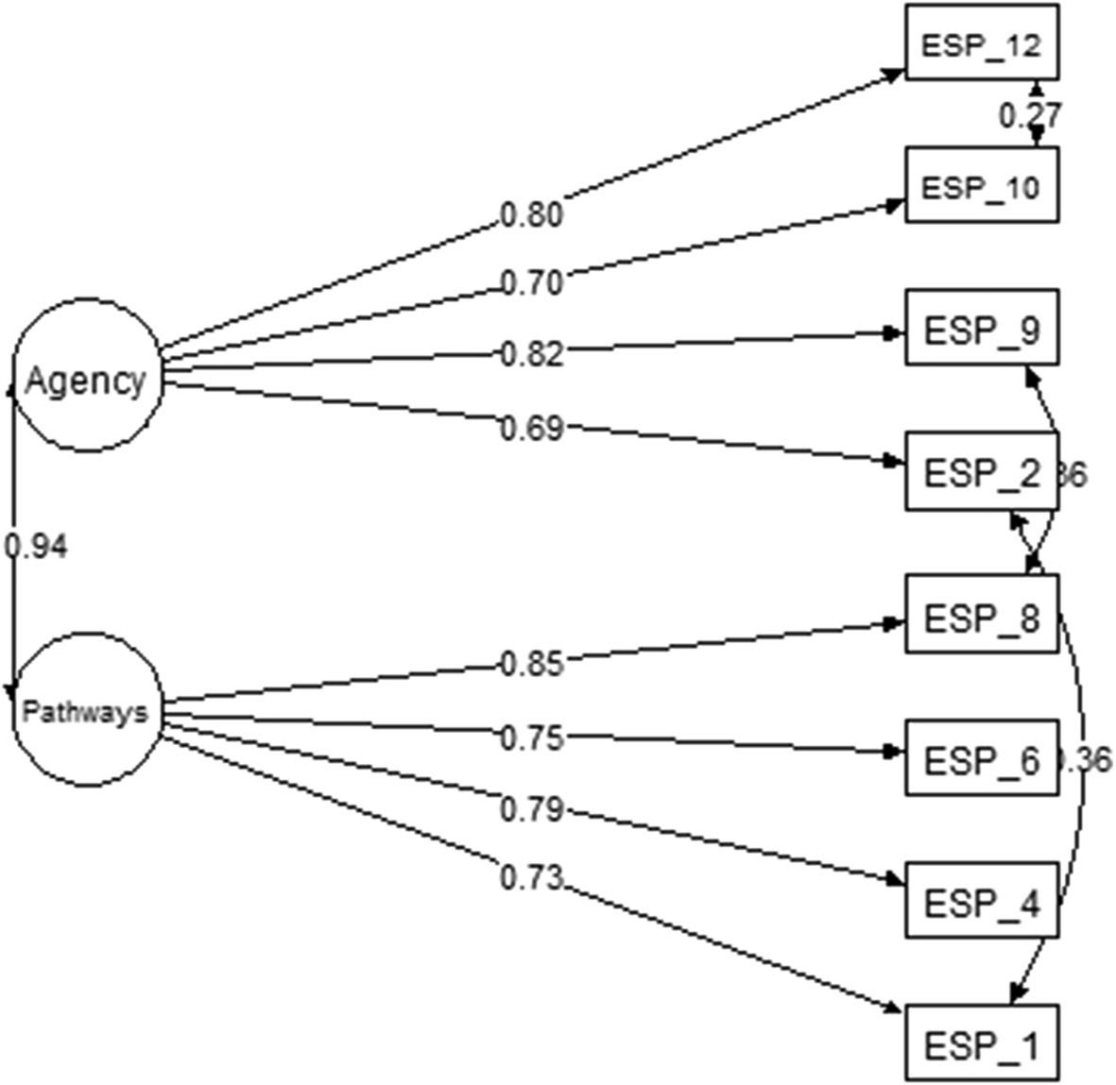
Two first-order oblique factors structural factor model for the ADHS

Model 4 assessed if ADHS was constituted by two first-order oblique factors. The correlated factor model comprehends that the phenomenon is constituted by two or more latent factors associated, in a way that the variance of each observed variable would be explained only by one latent factor. Also, the shared variance between the latent variables would be explained by their association (Brown, 2015; Dunn & McCray, 2020). In this model, it was evaluated the adequacy of a solution with agency and pathways associated. In the two first-order oblique factors structure (model 4), the pathways and agency were highly correlated (*r* = .94). Analysis of the modification indexes indicated the inclusion of error covariances for the following item pairs: 1-2 (MI = 99.76), 8-9 (MI = 66.68), and 10-12 (MI = 79.65). The model with correlated errors showed adequate goodness-of-fit indices without considering the value of *X*^2^/df, which was high (Table 2), and all items had factor loadings higher than .65 (Fig. 4). The high correlation between the factors proves that the two-first order oblique model is not adequate for ADHS. It also indicates that the association of agency and pathways could be quantified in a higher-order factor to measure hope.

To assess if the goodness-of-fit indices of models 1–4 were significantly different, a chi-square difference (Δ*χ*^2^) analysis was conducted. The results show that model 2 had significantly better goodness-of-fit indices than model 1 [(Δ*χ*^2^ = 348.1 (2), *p* < .001], model 3 [(Δ*χ*^2^ = 406.6 (7), *p* < .001], and model 4 [(Δ*χ*^2^ = 390.1 (3)]. Model 3 was equivalent to model 1 [(Δ*χ*^2^ = 108.9 (5), *p* > .001] and model 4 [(Δ*χ*^2^ = 190.6 (4), *p* < 0.001]. Finally, model 1 had significantly better goodness-of-fit indices than model 4 [(Δ*χ*^2^ = 44.1 (1), *p* < 0.001]. Although this analysis indicated the superiority of model 2, the high factorial loading of the dimensions agency and pathways showed that this solution was inadequate for the Ecuadorian ADHS.

Similarly, the item factorial loadings of model 4 demonstrate the limitations of the bifactor model to investigate a general factor of hope and the dimensions of agency and pathways. We suggest that the ADHS is adequate to investigate hope relationships and their impact in an Ecuadorian setting based on these results. Furthermore, based on goodness-of-fit indices, model parsimony, and item factorial loadings, we concluded that the unidimensional structure solution is the most adequate structure for the Ecuadorian version of the ADHS (Table 3 and Fig. 1).

**Table 3:** Gender (male × female) measurement invariance for ADHS

ADHS	*χ*^2^ (*gl*)	CFI	ΔCFI	RMSEA	ΔRMSEA
**Unconstrained model**	788.9* (40)	.946	–	.125 (.118–.133)	-
**Metric invariance**	793.6* (63)	.948	.002	.099 (.093–.010)	.026
**Scalar invariance**	4650.5* (70)	.958	.01	.083 (.078–.089)	.005
**Uniqueness invariance**	650.5* (70)	.958	.00	.083 (.078–.089)	.000

After identifying the most adequate structure of the Ecuadorian ADHS, multigroup CFA was performed to test the measurement invariance of the scale with respect to gender. According to the guidelines from Putnick and Bornstein (2016), the results suggest that the unidimensional ADHS achieved metric, scalar, and uniqueness invariance according to the ΔCFI and ΔRMSEA values between the genders (male and female, Table 3).

### Reliability analysis

The reliability index of the Ecuadorian version of the ADHS was excellent [ordinal *α* (95% CI) = .92 (.91–.92), *ϖ* (95% CI) = .87 (.85–.88), and CR = .95]. These results provide evidence that ADHS is a reliable instrument for assessing the general dimension of hope (Valentini & Damásio, 2016; Viladrich et al., 2017).

### Evidence of convergent and discriminant validity

We evaluated the evidence of convergent validity for the ADHS, assuming its unidimensionality. The results presented in Table 4 showed that dispositional hope is positively and strongly associated with work engagement. By contrast, hope is negatively associated with levels of burnout and its dimensions (exhaustion, mental distance, cognitive impairment, and emotional impairment), and these relationships have moderate magnitude. Work engagement is negatively related to burnout and its dimensions, with moderate to solid magnitudes. All variables showed AVE values ≥ .50, which is considered adequate for these structural models. Furthermore, the AVE values of the scales were more significant than their squared correlations (*r*^2^) with ADHS, demonstrating that there was discriminant validity between ADHS and work engagement and burnout and its dimensions. These results show that the measurement of hope by ADHS can be distinguished from the other variables evaluated in this study.

**Table 4:** Relationships between dispositional hope and work engagement and burnout and its dimensions

	M	SD	AVE	1	2	3	4	5	6
1) Hope	4.4	0.6	.57						
2) Work engagement	5.8	1.1	.71	.50** [.45, .51]					
3) Burnout	1.8	0.6	.97	− .36** [− .40, − .33]	− .59** [− .62, − .56]				
4) Exhaustion	2.2	0.8	.63	− .25** [− .29, − .21]	− .50** [− .53, − .47]	.88** [.87, .89]			
5) Mental distance	1.7	0.7	.58	− .32** [− .36, − .28]	− .50** [− .52, − .47]	.83** [.82, .84]	.62** [.60, .64]		
6) Cognitive impairment	1.5	0.6	.80	− .38** [− .41, − .35]	− .52** [− .55, − .49]	.83** [.82, .84]	.57** [.55, .60]	.64** [.61, .66]	
7) Emotional impairment	1.5	0.6	.73	− .35** [− .38, − .31]	− .49** [− .52, − .46]	.82** [.81, .84]	.57** [.54, .60]	.62** [.60, .65]	.75** [.73, .76]

*M* mean, *SD* standard deviation, *AVE* average variance extracted

**p < 0.01

## Discussion

Ecuador’s cultural and social particularities (Merz et al., 2011; Ravallion, 2014; Vinueza-Solórzano et al., 2021) motivated the development of the study of the psychometric properties of the Ecuadorian version of the ADHS. Furthermore, this study added evidence to the debate on the factor structure of the ADHS (Alfieri et al., 2021; Brouwer et al., 2008; Gomez et al., 2015; Roesch & Vaughn, 2006), so the first aim of our study was to adapt the ADHS (Snyder et al., 1991) and obtain evidence of its validity for use specifically in Ecuador.

The lack of agreement on the best structure for the ADHS impacts the interpretation of ADHS scores in a Spanish-speaking population, especially considering the scarcity of studies that have evaluated the psychometric properties of the ADHS for these populations (Galiana et al., 2015; Querido & dos Anjos Dixe, 2016). The present study also assesses the goodness-of-fit indices of four structures for the ADHS: unidimensional, second-order, bifactor, and two first-order oblique factor models.

Our results show that the ADHS is valid for measuring dispositional hope in Ecuadorian individuals. In particular, the unidimensional ADHS structure has excellent dimensionality, reliability, and validity evidence. For the Ecuadorian context, we recommend evaluating the general factor of hope rather than differentiating the impact of agency and pathways on the measurement of levels of hope or their relationship with other variables.

### The psychometric properties of the ADHS Ecuadorian version

The results of the dimensionality of the ADHS Ecuadorian version show that the unidimensional structure was the most adequate. We decided to adopt the unidimensional structure for the ADHS at the expense of the two first-order oblique factors (Alfieri et al., 2021; Brouwer et al., 2008; Roesch & Vaughn, 2006) and the two first-order factors with a second-order factor model. The evaluation of the two first-order oblique factors model evidence that pathways and agency shared 92% of their variance, consistent with the findings of Gomez et al. (2015). Also, the results of the second-order model of the ADHS Ecuadorian version showed that the association between the first-order factors and the second-order factor was almost 1.00, indicating that the three latent variables shared a high percentage of the explained variance. The high value of shared variance between the factors makes it challenging to identify the specific contribution of each dimension (Fornell & Larcker, 1981). The unidimensional model was considered the best solution for the ADHS Ecuadorian version based on these findings.

In the bifactorial model, the S-factors should explain part of the variance that was not explained by the G-factor (Dunn et al., 2014; Reise et al., 2010). As expected, the results show that most of the agency’s items and pathways’ items (S-factor) share their variance with the dimension hope (G-factor). Also, all items presented a higher factor loading in the hope dimension than the agency and pathways dimensions. The findings indicate that the G-factor was responsible for explaining most of the variance of the observable variables. Therefore, our results prove that the bifactor structure of two oblique S-factors (agency and pathways) and one G-factor (hope) is not optimal for ADHS.

For models 1 and 4, modification indexes suggested the inclusion of error covariances for the item pairs 1-2, 8-9, and 10-12. Both items 1 (“I can think of many ways to get out of a jam.”) and 2 (“I energetically pursue my goals.”) refer to planning and acting toward personal goals and searching for a positive state of mind. Items 8 (“Even when others get discouraged, I know I can find a way to solve the problem.”) and 9 (“My past experiences have prepared me well for my future.”) aim to evaluate self-perceptions about individuals to overcome adversities. Items 10 (“I have been pretty successful in life.”) and 12 (“I meet the goals that I set for myself.”) aim to evaluate self-perceptions about individuals’ achievements and abilities to conquer their personal goals. All items with correlated errors included content focusing on individual abilities, planning and actions toward their aims, and overcoming adversities. The overlapping content of these pairs of items might explain the residual correlation between the items.

This assessment of the general factor hope is aligned with the recommendations of Snyder et al. (1991), who suggested that the amount of variance shared by agency and pathways indicates that these dimensions measure the same construct and that there is a slightly single variance that is explained by the pathways or agency items above the general factor hope. The unidimensional structure of the ADHS also serves the practical purpose of probing the general contributions of dispositional hope into an occupational context, which is in the present days considered under crisis, due to the conditions created by the COVID-19 pandemic and sudden changes experienced in working settings (Kniffin et al., 2021). Using the total score of hope, it is possible to look at the underlying relevancy of the hope factor to other external work-related variables and its various protective roles in mental health and occupational well-being (Chang, 2003; Passmore et al., 2020; Yotsidi et al., 2018).

The results evidence the excellent reliability scores of the ADHS Ecuadorian version. These findings confirm hypothesis 2 that the unidimensional solution would be an adequate structure for the scale and reinforce that this is a reliable tool to evaluate dispositional hope in the Ecuadorian context. Furthermore, the Ecuadorian version of ADHS was invariant for gender, indicating that ADHS is a robust scale to assess hope levels in both women and men.

### Relations of hope, work engagement, and burnout

The evidence of convergent validity showed that dispositional hope obtained a positive association with work engagement, evidencing that dispositional hope may promote higher levels of positive work well-being states (Hofkamp et al., 2020; Sarfraz et al., 2019). Also, following hypothesis 4, dispositional hope was negatively associated with burnout and its dimensions, showing that higher levels of hope may act as a protective factor to prevent the development of burnout (Hofkamp et al., 2020; Passmore et al., 2020; Yotsidi et al., 2018). The protective role that dispositional hope plays in the context of the professionals’ work shows that it is characterized as a personal resource that contributes to planning their professional goals and driving their actions to achieve them (Snyder et al., 1991). Future studies should focus on the impact of hope associated with social support due to Ecuador’s collectivist values (Merz et al., 2011; Ravallion, 2014; Vinueza-Solórzano et al., 2021).

### Limitations and future directions

Regarding the study’s limitations, using a nonrepresentative sample might make complex, deeper characterization of the impact of adult dispositional hope on specific professional groups or individuals with different socio-demographic features. Further studies on different samples (e.g., professors, students, healthcare workers) with various sociodemographic features (e.g., age, gender, educational level) are needed to investigate the stability of the unidimensional structure of ADHS and compare the level of impact that the dispositional hope has between different groups in variables identified as outcomes of hope, such as work engagement and burnout.

## Conclusion

This study’s unique and robust character derives from the analysis of four structural models of the Ecuadorian version of the ADHS, as such studies are scarce in the literature (Galiana et al., 2015; Pleeging et al., 2021). Furthermore, the robustness of the results is enhanced by the adoption of data analysis procedures that apply corrections for the characteristics of ordinal and non-scalar variables. The research’s main strength lies in the conditions created globally by the COVID-19 pandemic, in which severe health consequences have developed for people in different areas, especially those who work. This emphasizes monitoring stressor processes, considering the importance of protective factors in their contexts, and adding new variables into a contextual analysis. Our study, and the Ecuadorian version of the ADHS, is a broad contribution to the professionals dealing with this topic in the field in Ecuador and those who will adopt this tool now have a means for developing novel and more informed and integrated hope-based approaches to help individuals, especially the working population, to deal with the demands of life and mitigate risks to their well-being.

## Data Availability

The datasets used and analyzed during the current study are available from the corresponding authors upon reasonable request.

## Abbreviations

ADHS: Adult Dispositional Hope Scale
HHI: Hert Hope Index
PPAM: Positive Psychological Assessment Measures
SD: Standard deviation
BAT: Burnout Assessment Tool
CFI: Comparative Fit Index
TLI: Tucker-Lewis Index
RMSEA: Root mean square error of approximation
CR: Composite reliability
AERA: American Educational Research Association
APA: American Psychological Association
NCME: National Council on Measurement in Education
WLSMV: Weighted least squares means and variance adjusted
CFA: Confirmatory factorial analysis
MI: Modification indices
AVE: Average variance extracted
Df: Degrees of freedom
CI: Confidence interval/cognitive impairment
M: Mean
H: Hope
WE: Work engagement
B: Burnout
Ex: Exhaustion
MD: Mental distance
EI: Emotional impairment
